# The reliability of a Biometrics device as a tool for assessing hand grip and pinch strength, in a Polish cohort–A prospective observational study

**DOI:** 10.1371/journal.pone.0303648

**Published:** 2024-05-23

**Authors:** Justyna Leszczak, Bogumiła Pniak, Mariusz Drużbicki, Agnieszka Guzik

**Affiliations:** 1 Institute of Health Sciences, Medical College, University of Rzeszów, Rzeszów, Poland; 2 Excelsior Health and Rehabilitation Hospital, Iwonicz-Zdrój, Poland; Aichi Prefectural Mikawa Aoitori Medical and Rehabilitation Center for Developmental Disabilities, JAPAN

## Abstract

The aim of the study was to assess the external and internal compatibility of the Biometrics E-LINK EP9 evaluation system device in the area of hand grip and pinch strength in the Polish population. The testing of hand grip and pinch strength was carried out among 122 healthy students. Two examiners performed hand grip and pinch strength measurements with a Biometrics E-LINK EP9 evaluation system device. Measurements were made for the right and left hands. The same people were tested again two weeks later, under the same conditions. The scores of one rater on the first and second tests were compared for reproducibility, and the scores of the two raters were compared to assess the reliability of the instrument. The measurements were found to be highly consistent both between the investigators and between the tests in the hand grip dynamometer test. The findings show high values of the Pearson’s correlation coefficient equal or close to 1, as well as the interclass correlation coefficient (ICC) >0.9. Analysis of pinch strength measurements performed using the pinchmeter also found high values of the Pearson’s correlation coefficient close to 1, as well as the interclass correlation coefficient >0.9; this reflects high agreement between the measurements performed by two investigators as well as assessments performed by one investigator at time intervals. These findings were confirmed by analyses performed using Bland-Altman plots. The measurements made with the Biometrics E-link EP9 evaluation system show high internal and external consistency in hand grip and pinch strength assessment. Biometrics E-link EP9 can be recommended for daily clinical practice.

## Introduction

Hand grip and finger grip strength measurements are important parameters for assessing hand function [1]. Grip strength can be easily measured, and, on the basis of studies, it has been found to be associated with musculo-skeletal diseases of the upper limbs [2]. Grip strength is an important feature in the processes of personal development, ageing, training, as well as injury and rehabilitation. [1,3]. Numerous clinical and epidemiological studies have shown the prognostic potential of hand grip for short- and long-term morbidity and mortality [4–8]. Studies have shown that low grip strength in healthy adults predicts an increased risk of functional limitations and disability in older age [4–12].

The measurement of grip strength is very useful in clinical areas, including occupational medicine, sports medicine, orthopaedics, rheumatology and neurology [2]. Spherical hand grip strength is clinically relevant and is evaluated by physicians and physiotherapists in the assessment and comparison of operating techniques, observation of rehabilitation effects and assessment of the degree of disability after injury. Hand grip strength is also used to assess the performance of athletes who rely on the right level of grip strength to increase control and performance and minimise possible injuries [13,14].

In order to make sure that hand and finger grip strength measurements are clinically useful, their credibility, reliability and consistency must be confirmed [15,16], since valid and reliable assessment tools are essential for patient treatment, professional communication and development [17]. The literature related to this subject matter discusses a variety of isometric and isokinetic tools, as well as hand-held piezoelectric tools for assessing hand grip strength, such as e.g. Lido WorkSe, Biodex System 3 or gloves used with Southampton Intelligent Hand [18–21]. Likewise, in addition to a Jamar hydraulic dynamometer, assessment of hand grip strength is performed using other devices, such as DynEX, Grip-ball, Smedley and others [15,22,23]. However, it is the Jamar dynamometer that is considered to be the gold standard for measuring grip strength and is recommended by the American Society of Hand Therapists (ASHT) [24–28]. Many researchers have used the Jamar dynamometer as a standard for validation of other dynamometers [29–36], including the Biometrics dynamometer. Allen and Barnett in Australia used the Jamar dynamometer as a standard to assess grip strength to evaluate the reliability and validity of the Biometrics E-link EP9 dynamometer. The assessment was conducted in a group of forty-nine students aged 18 to 25 years. The study showed that the Biometrics E-link EP9 dynamometer is reliable, valid and comparable to the Jamar hydraulic dynamometer [37]. Despite the evidence showing that Biometrics E-link EP9 dynamometer can reliably be used to measure hand grip strength, Allen and Barnett’s study presented a significant limitation resulting from the small sample size (49 participants, including only 7 male participants). To amend for this drawback, the present study was designed to involve a significantly larger group. Furthermore, we assessed three types of pinch strength (key, three jaw chuck, tip-to-tip) which were not investigated by Allen and Barnett. Another interesting study, conducted by Huang et al., took into account 1,064 adults, including 772 senior citizens with chronic conditions which are likely to affect hand grip strength. Because of this the latter authors suggested it would be worthwhile to assess hand grip in a population of young individuals with no medical conditions or comorbidities [38]. This recommendation was also taken into account in the current study.

The scientific literature related to these issues suggests that the Biometrics E-LINK dynamometer and pinchmeter are used around the world, both for the assessment of daily activities, muscle activity, functional status, and to measure the effects of rehabilitation or surgical interventions [39–49], both in healthy participants [39] and patients with various upper limb problems associated with: hand prosthesis [40], spinal cord injury [41], stroke [42], distal radius fractures [43], osteoarthritis [44] multiple sclerosis [45], chronic lower back pain [46], tennis elbow [47] carpal tunnel release [48], heart disease and pulmonary hypertension [49]. However, despite such multi-faceted use of this device, there are few reports in the field of inter- and intra-rater reliability in the field of hand grip and pinch strength measurement using the Biometrics E-link [37,50].

This observation was further motivation to undertake this research, which is the first report on this issue in Poland. Therefore, the aim of the study was to assess the consistency of the Biometrics external and internal electronic dynamometer in the field of hand grip and pinch strength in the Polish population.

## Materials and methods

### Study design

The research was conducted as a prospective observational study. It was carried out in accordance with the ethical rules of the Helsinki Declaration, and approved by the local bioethics commission (consent no. 2022/036/W). Written informed consent was obtained from all participants in the study.

### Setting

The study was conducted at the Laboratory of Innovative Biofeedback Methods of the University of Rzeszów.

### Participants

The data were collected from 1 August 2022 to 28 February 2023. The study included healthy individuals who gave their voluntary informed consent to participate; they were physiotherapy students, aged 20–24 years, without dysfunction in the upper limbs, which was confirmed by medical examination. The study excluded people with upper limb dysfunctions, i.e. after injuries (dislocation, sprains, fractures), burns, with contractures, with pathological muscle tension, with muscular atrophy, or with neurological, orthopaedic or rheumatoid diseases affecting the function of the upper limbs.

### Sample size

Before starting the research, the sample size was calculated based on the annual number of students in the field of Physiotherapy at the University of Rzeszów in Poland. The sample size was calculated for a 95% confidence interval, a fraction sizeof 0.08 and a maximum error of 6%. Using a sample selection calculator, we obtained a minimum sample size of 97 people, and a sample larger than the minimum sample size was finally qualified for the study, i.e. 122 people.

### Procedure

Hand grip and finger grip strength were assessed using the Biometrics E-link EP9 electronic dynamometer and pinchmeter. The dynamometer records forces from less than 0.1kg/lb up to 90 kg (200lbs), and the pinchmeter up to 22kg (50lbs) [37,51]. The standard grip test was performed three times for each hand that was analysed. The pinchmeter was also measured three times for each hand with three different settings (key, three jaw chuck, tip-to-tip). All measurements were made by two independent examiners, at the same time and under the same conditions. In order to ensure that the measurements were as reliable as possible, the examiners did not communicate with each other.

**Fig 1:**
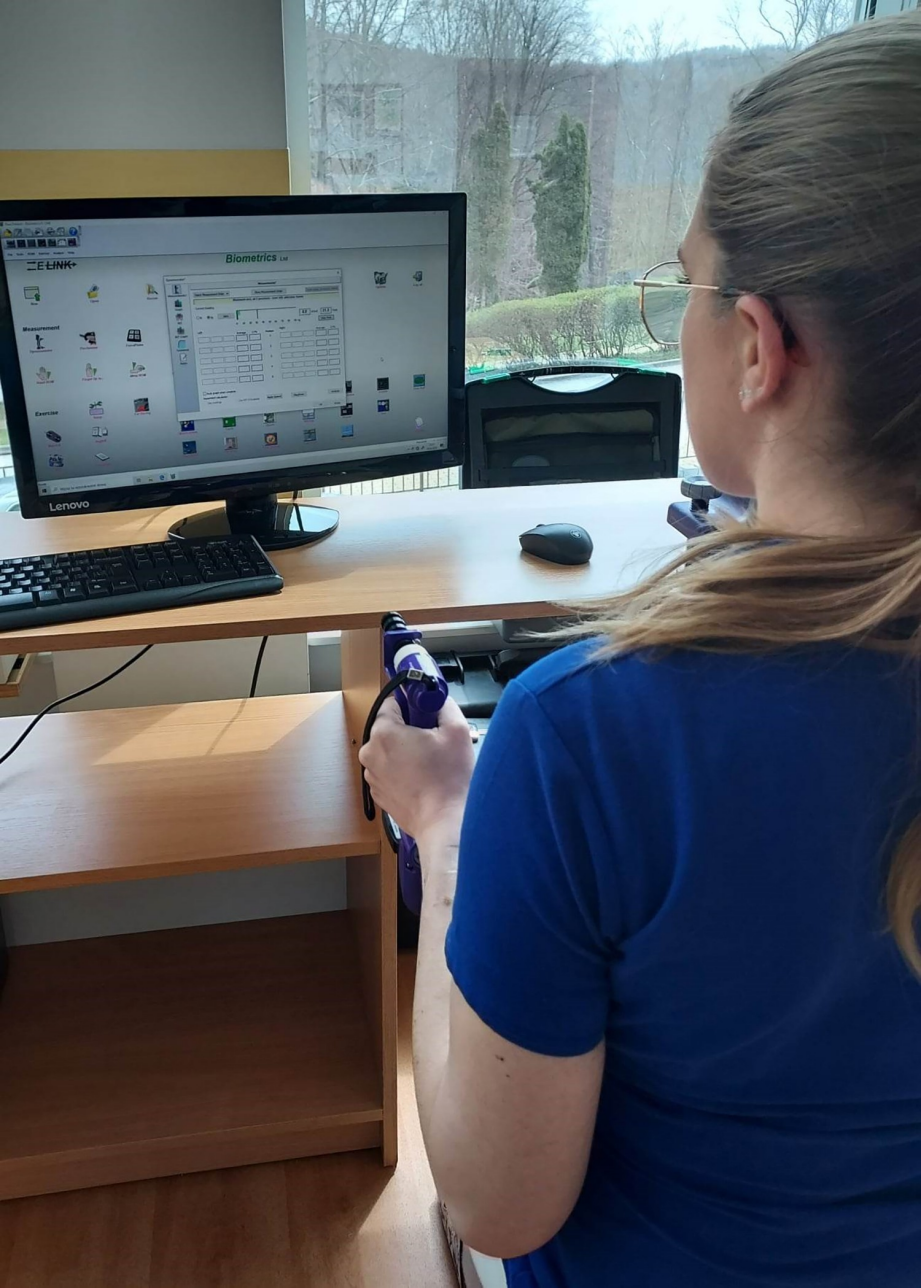
Biometrics E-LINK EP9 hand grip dynamometer.

Tests of hand grip strength and finger grip strength were conducted in accordance with the American Society of Hand Therapists (ASHT): shoulders adducted and neutrally rotated, elbow flexed to 90°, forearm in neutral position and wrist between 0–30° of extension and 0–15° of ulna deviation, and feet flat on the floor [52]. Participants maintained this position throughout the testing process. The respondents received an oral command, which was: “First we will test your left hand. You will have to grip the dynamometer as hard as possible and then hold it for a count of three: one, two, three.” The procedure was repeated with the right hand. And then the same measurements were taken for finger grip: key, three jaw chuck, tip-to-tip (Fig 1). Intervals of 15 seconds were applied between the trials to avoid muscle fatigue. The first test was followed by a 5-minute break and then assessments were carried out to measure grip strength and three types of pinch strength in the same manner, and the respective average strengths were obtained. The length of the break was determined in line with the manufacturer’s recommendations. On the other hand, the duration of each contraction was three seconds [37,51]. No adverse events were observed during the study. The authors had access to personally identifiable information for individual participants during or after data collection as the authors were also with the investigators.

An analysis of external and internal consistency was carried out, i.e. the repeatability of measurements made by the two investigators at the same time, and by the same investigator at two times, i.e. two weeks apart. In total, therefore, four comparisons of two data series were made for each parameter.

### Statistical methods

The analysis concerned a homogeneous group of students, they were people of similar age and healthy. The mean and standard deviation for each series of measurements were calculated, along with the mean and standard deviation of the differences between the series of measurements compared. The significance of the differences in the average level of two series of measurements was assessed using the t-test for dependent Between the compared series, Pearson’s linear correlation coefficient was used, together with a key measure of the consistency of two measurements–the interclass correlation coefficient (ICC). As an alternative measure of consistency, Cronbach’s alpha coefficient as well as Bland-Altman’s plots were applied. The level of statistical significance was p<0.05.

## Results

**Fig 2:**
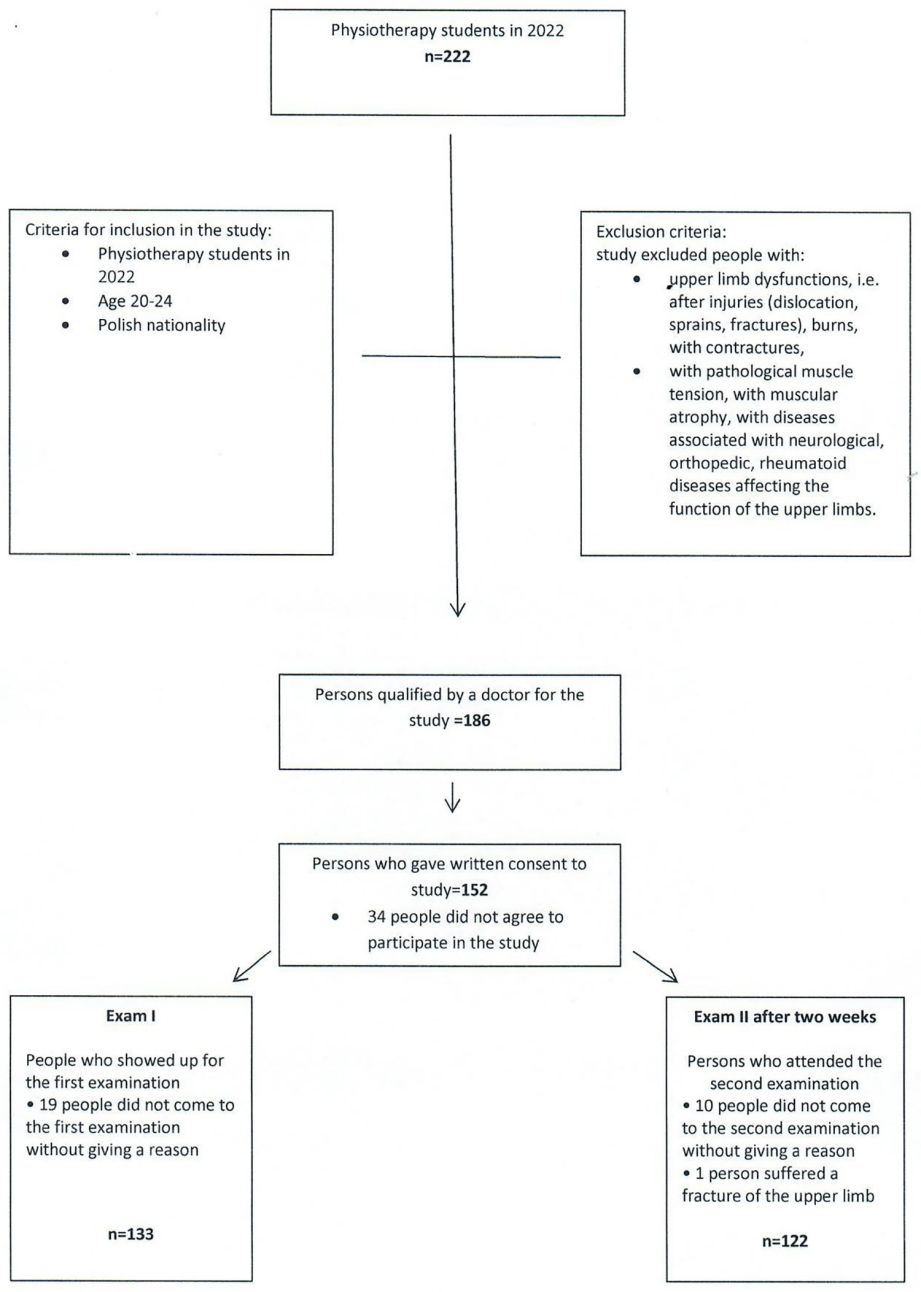
Flowdiagram.

Using the sample selection calculator, we obtained a minimum sample size of 97 people, and a sample larger than the minimum sample size was finally qualified for the study, i.e. 122 people, who were selected from 222 students, as follows: out of 222 Physiotherapy students in 2022, 186 people were qualified for the study on the basis of inclusion and exclusion criteria and a medical examination. Thirty-four individuals did not give written consent to participate in the study. Written consent was obtained from 152 people, of whom 19 did not report for examination I without giving a reason. 133 people took part in the first examination. 10 people did not come to the second examination without giving a reason and 1 person suffered a fracture of the upper limb. Two studies were conducted among 122 people. (Fig 2).

Mean age of the respondents was 22.2 ± 1.46 years. In the study group there were 44 men and 78 women. Mean body mass of the participants was 76.3 ± 12.64 kg and mean height of the participants was 170.1 ± 7.48 cm. The characteristics of the study group are shown in Tables 1 and 2.

**Table 1:** Gender distribution of the study group.

Sex	N	N Cumulative	Percentage
**Women**	78	78	63.93
**Men**	44	122	36.07

**Table 2:** Descriptive statistics–age, body height [m], body mass [kg].

Variables	Descriptive statistics							
	N	$\bar{x}$	Me	Min.	Max.	Q1	Q3	*SD*
**Age [years]**	122	22.2	22.0	20.0	24	21	24	1.46
**Body height [m]**	122	76.3	75.0	49.0	128	67	85	12.64
**Body mass [kg]**	122	170.1	170	152	190	165	174	7.48

N—numbers of participants, Max.—maximum value, Me—median, Min—minimum value, SD—standard deviation, $\bar{x}$-mean; Q1: first quartile; Q3: third quartile.

### The consistency of measurements between investigators and between tests for hand grip

The analysis of the consistency of the measurements with a dynamometer for both the right and left hand showed high Pearson correlation coefficient values of 1 and close to 1, an interclass correlation coefficient (ICC) of >0.9, and taking into account alternative measurements of consistency with Cronbach alpha coefficient of >0.9 this indicates a high consistency of measurements between both investigators as well as tests performed by the same investigator at a time interval. See Table 3 for details (Table 3).

**Table 3:** The consistency of measurements between investigators and between tests for measuring the strength of the right hand and the left hand using a dynamometer.

Dynamometer HR—Right hand													
Test	Investigator	Measurements				Differences				*p*	*r*	ICC	*Cronbach’s alpha*
		$\bar{x}$	*SD*	*Me*	*s* ^*2*^	$\bar{x}$	*SD*	*Me*	*s* ^*2*^				
**I**	**1**	35.36	11.69	35.05	136.61	-0.21	1.06	-0.45	1.12	0.0292	1.00	0.996 (0.994–0.997)	0.998
**I**	**2**	35.58	11.66	32.20	136.00								
**II**	**1**	35.44	11.77	31.85	138.57	0.28	1.27	0.35	1.62	0.0172	0.99	0.994 (0.991–0.996)	0.997
**II**	**2**	35.72	11.70	32.20	136.92								
**I**	**1**	35.36	11.69	35.05	136.61	-0.08	1.10	-0.15	1.20	0.4396	1.00	0.996 (0.994–0.997)	0.998
**II**	**1**	35.44	11.77	31.85	138.57								
**I**	**2**	35.58	11.66	32.20	136.00	-0.14	1.06	0.00	1.13	0.1361	1.00	0.996 (0.994–0.997)	0.998
**II**	**2**	35.72	11.70	32.20	136.92								
**Dynamometer HL—Left hand**													
**I**	**1**	30.47	9.89	27.35	97.83	0.41	1.27	0.30	1.60	0.0006	0.99	0.991 (0.986–0.994)	0.996
**I**	**2**	30.06	9.87	26.50	97.48								
**II**	**1**	30.50	9.81	27.55	96.17	0.50	0.99	0.45	0.99	0.0000	0.99	0.994 (0.987–0.996)	0.997
**II**	**2**	30.01	9.68	26.80	93.77								
**I**	**1**	30.47	9.89	27.35	97.83	-0.04	0.84	-0.05	0.70	0.6352	1.00	0.996 (0.995–0.997)	0.998
**II**	**1**	30.50	9.81	27.55	96.17								
**I**	**2**	30.06	9.87	26.50	97.48	0.05	1.01	0.00	1.02	0.5556	0.99	0.995 (0.992–0.996)	0.997
**II**	**2**	30.01	9.68	26.80	93.77								

$\bar{x}$- mean; *SD*–standard deviation; *Me*–median; *s*^*2*^—a measure of variance; *p*–assessment of the significance of differences in the average level of two series of measurements (t-test for dependent samples); *r*–Pearson linear correlation coefficient; ICC–interclass correlation coefficient with 95% confidence interval; *Cronbach’s alpha*–alternative consistency measure.

### The consistency of measurements between investigators and between tests for pinch strength

The analysis of the consistency of the measurements using a pinchmeter, for both the right and left hand and for all three settings (key, three jaw chuck, tip-to-tip), showed high values of Pearson correlation coefficient close to 1, interclass correlation coefficient (ICC) of >0.9, and taking into account alternative measurements of consistency with Cronbach alpha coefficient of >0.9 this indicates a high consistency of measurements between both investigators as well as tests performed by the same investigator at a time interval. See Tables 4, 5 and 6 for details (Tables 4, 5 and 6).

**Table 4:** The consistency of measurements between investigators and between tests for measuring the strength of the fingers of the right hand and the left hand using a pinchmeter (key).

Pinchmeter HR—Right hand (key)													
Test	Investigator	Measurements				Differences				*p*	*r*	ICC	*Cronbach’s alpha*
		$\bar{x}$	*SD*	*Me*	*s* ^*2*^	$\bar{x}$	*SD*	*Me*	*s* ^*2*^				
**I**	**1**	8.17	1.85	7.90	3.43	0.17	0.32	0.20	0.10	0.0000	0.99	0.981 (0.959–0.990)	0.992
**I**	**2**	8.00	1.80	7.75	3.23								
**II**	**1**	7.95	1.81	7.70	3.26	0.03	0.28	0.10	0.08	0.2895	0.99	0.987 (0.982–0.991)	0.994
**II**	**2**	7.92	1.74	7.60	3.04								
**I**	**1**	8.17	1.85	7.90	3.43	0.22	0.36	0.20	0.13	0.0000	0.98	0.974 (0.934–0.987)	0.990
**II**	**1**	7.95	1.81	7.70	3.26								
**I**	**2**	8.00	1.80	7.75	3.23	0.08	0.36	0.10	0.13	0.0225	0.98	0.979 (0.969–0.985)	0.990
**II**	**2**	7.92	1.74	7.60	3.04								
**Pinchmeter HL—Left hand (key)**													
**I**	**1**	7.16	1.63	6.90	2.66	0.16	0.17	0.20	0.03	0.0000	0.99	0.990 (0.939–0.996)	0.997
**I**	**2**	7.00	1.60	6.70	2.57								
**II**	**1**	7.01	1.65	6.70	2.73	0.03	0.24	0.10	0.06	0.1820	0.99	0.989 (0.984–0.992)	0.994
**II**	**2**	6.98	1.60	6.75	2.57								
**I**	**1**	7.16	1.63	6.90	2.66	0.15	0.23	0.20	0.05	0.0000	0.99	0.986 (0.957–0.994)	0.995
**II**	**1**	7.01	1.65	6.70	2.73								
**I**	**2**	7.00	1.60	6.70	2.57	0.02	0.21	0.00	0.05	0.2222	0.99	0.991 (0.987–0.994)	0.996
**II**	**2**	6.98	1.60	6.75	2.57								

$\bar{x}$- mean; *SD*–standard deviation; *Me*–median; *s*^*2*^—a measure of variance; *p*–assessment of the significance of differences in the average level of two series of measurements (t-test for dependent samples); *r*–Pearson linear correlation coefficient; ICC–interclass correlation coefficient with 95% confidence interval; *Cronbach’s alpha*–alternative consistency measure.

**Table 5:** The consistency of measurements between investigators and between tests for measuring the strength of the fingers of the right hand and the left hand using a pinchmeter (three jaw chuck).

Pinchmeter HR—Right hand (three jaw chuck)													
Test	Investigator	Measurements				Differences				*p*	*r*	ICC	*Cronbach’s alpha*
		$\bar{x}$	*SD*	*Me*	*s* ^*2*^	$\bar{x}$	*SD*	*Me*	*s* ^*2*^				
**I**	**1**	7.63	1.53	7.50	2.35	0.16	0.19	0.10	0.04	0.0000	0.99	0.987 (0.937–0.995)	0.996
**I**	**2**	7.47	1.51	7.40	2.29								
**II**	**1**	7.52	1.50	7.40	2.26	0.09	0.23	0.10	0.05	0.0001	0.99	0.986 (0.977–0.991)	0.994
**II**	**2**	7.43	1.51	7.35	2.27								
**I**	**1**	7.63	1.53	7.50	2.35	0.11	0.22	0.10	0.05	0.0000	0.99	0.987 (0.975–0.993)	0.995
**II**	**1**	7.52	1.50	7.40	2.26								
**I**	**2**	7.47	1.51	7.40	2.29	0.04	0.22	0.10	0.05	0.0667	0.99	0.989 (0.985–0.992)	0.995
**II**	**2**	7.43	1.51	7.35	2.27								
**Pinchmeter HL—Left hand (three jaw chuck)**													
**I**	**1**	6.84	1.39	7.00	1.93	0.13	0.15	0.10	0.02	0.0000	0.99	0.990 (0.940–0.996)	0.997
**I**	**2**	6.71	1.37	6.80	1.88								
**II**	**1**	6.74	1.37	6.80	1.87	0.05	0.26	0.05	0.07	0.0495	0.98	0.982 (0.975–0.988)	0.991
**II**	**2**	6.69	1.39	6.70	1.93								
**I**	**1**	6.84	1.39	7.00	1.93	0.11	0.19	0.10	0.04	0.0000	0.99	0.988 (0.972–0.993)	0.995
**II**	**1**	6.74	1.37	6.80	1.87								
**I**	**2**	6.71	1.37	6.80	1.88	0.02	0.25	0.00	0.06	0.4283	0.98	0.984 (0.977–0.988)	0.992
**II**	**2**	6.69	1.39	6.70	1.93								

$\bar{x}$- mean; *SD*–standard deviation; *Me*–median; *s*^*2*^—a measure of variance; *p*–assessment of the significance of differences in the average level of two series of measurements (t-test for dependent samples); *r*–Pearson linear correlation coefficient; ICC–interclass correlation coefficient with 95% confidence interval; *Cronbach’s alpha*–alternative consistency measure.

**Table 6:** The consistency of measurements between investigators and between tests for measuring the strength of the fingers of the right hand and the left hand using a pinchmeter (tip-to-tip).

Pinchmeter HR—Right hand (tip to tip)													
Test	Investigator	Measurements				Differences				*p*	*r*	ICC	*Cronbach’s alpha*
		$\bar{x}$	*SD*	*Me*	*s* ^*2*^	$\bar{x}$	*SD*	*Me*	*s* ^*2*^				
**I**	**1**	6.18	1.24	6.30	1.54	0.14	0.15	0.10	0.02	0.0000	0.99	0.987 (0.897–0.995)	0.996
**I**	**2**	6.04	1.22	6.20	1.50								
**II**	**1**	6.06	1.21	6.20	1.48	0.05	0.19	0.10	0.04	0.0100	0.99	0.987 (0.981–0.991)	0.994
**II**	**2**	6.02	1.22	6.10	1.49								
**I**	**1**	6.18	1.24	6.30	1.54	0.12	0.19	0.10	0.04	0.0000	0.99	0.984 (0.957–0.992)	0.994
**II**	**1**	6.06	1.21	6.20	1.48								
**I**	**2**	6.04	1.22	6.20	1.50	0.02	0.17	0.00	0.03	0.1328	0.99	0.991 (0.986–0.993)	0.995
**II**	**2**	6.02	1.22	6.10	1.49								
**Pinchmeter HL—Left hand (tip to tip)**													
**I**	**1**	5.52	1.19	5.35	1.41	0.14	0.16	0.20	0.03	0.0000	0.99	0.984 (0.926–0.994)	0.995
**I**	**2**	5.38	1.19	5.20	1.41								
**II**	**1**	5.37	1.20	5.30	1.43	-0.02	0.22	-0.05	0.05	0.3292	0.98	0.983 (0.976–0.988)	0.991
**II**	**2**	5.39	1.20	5.25	1.44								
**I**	**1**	5.52	1.19	5.35	1.41	0.14	0.20	0.20	0.04	0.0000	0.99	0.978 (0.929–0.990)	0.993
**II**	**1**	5.37	1.20	5.30	1.43								
**I**	**2**	5.38	1.19	5.20	1.41	-0.01	0.21	0.00	0.04	0.4877	0.98	0.985 (0.978–0.989)	0.992
**II**	**2**	5.39	1.20	5.25	1.44								

$\bar{x}$- mean; *SD*–standard deviation; *Me*–median; *s*^*2*^—a measure of variance; *p*–assessment of the significance of differences in the average level of two series of measurements (t-test for dependent samples); *r*–Pearson linear correlation coefficient; ICC–interclass correlation coefficient with 95% confidence interval; *Cronbach’s alpha*–alternative consistency measure.

The results of strength measurements shown by Bland-Altman plots indicate that the highest level of agreement was observed in the case of the first investigator between Test 1 and Test 2 (for both right and left hand). Mean deviations of only 0.01–0.04 kg were found, and the deviations of individual values in most cases did not exceed ±2 kg (S1 and S2 Figs).

The Bland-Altman plots show that the measurements of finger strength in the right hand were most consistent in Test 2 results, when compared between the first and the second investigator, with the difference in the mean values amounting to 0.03 kg. Furthermore, in measurements of the left hand the highest agreement was observed between Test 1 and Test 2 performed by the second investigator, with the difference between the mean values amounting to 0.02 kg (S3 and S4 Figs).

The highest agreement in measurements of finger strength in the three-jaw chuck test was found between Test 1 and Test 2 performed by the second investigator. Mean deviations of only 0.04 and 0.02 kg were found for the right and the left hand, respectively, and the deviations of individual values in most cases did not exceed ±0.5 kg (S5 and S6 Figs).

Similarly, in the measurements of finger strength in the tip-to-tip test, the most consistent values were observed in the case of the second investigator in the two tests. The differences in the mean values for the right and the left hand were 0.02 and -0.01 kg, respectively. A vast majority of the measurements deviated by ±0.3 kg (S7 and S8 Figs).

## Discussion

The existing scientific literature includes reports related to assessment of wrist position sense [53] and ranges of motion in hands and forearms [54]. These are important and useful measurements; however, the aim of this study was to assess the consistency of the Biometrics E-link EP9 external and internal evaluation system in measuring hand grip and pinch strength in a Polish population. The study showed that the Biometrics E-link EP9 evaluation system has high internal and external consistency in the assessment of both hand grip and pinch strength.

There are currently few reports in the world literature presenting evidence that shows that the Biometrics E-link dynamometer is reliable, valid and comparable with the Jamar hydraulic dynamometer (recognised as the gold standard in the assessment of hand grip strength), as reflected by measurements carried out in a population of healthy individuals [37]. The excellent reliability of the tool was also shown in measurements of grip and pinch strength in a population of children with unilateral cerebral palsy [50]. The former study, however, was conducted in a small sample of healthy individuals, and assessed hand grip strength but not pinch strength [37]. This observation was the motivation to undertake this study.

Another motivation was the fact that the Biometrics E-link dynamometer and pinchmeter are widely used worldwide, not only with healthy individuals, but also patients with various upper limb dysfunctions, as mentioned above. However, despite the wide ranging use of the device, there is a paucity of research on inter- and intra-rater reliability of the tool in measurement of hand grip and pinch strength [37,50,55]. Furthermore, some researchers postulate that studies designed to evaluate the reliability and validity of the device should be conducted in groups of young people with no disorders or coexisting conditions which affect hand grip strength [38].

The results of our own research can only be compared with the single currently available report for healthy individuals, from Australia, and only in the area of the Biometrics E-link EP9 evaluation system [37]. Allen and Barnett in their research attempted to establish the reliability and validity of a Biometrics E-link EP9 electronic dynamometer for measuring grip strength. The grip strength test was performed on 49 healthy participants. Three tests were carried out for the right and left hand on Biometrics and Jamar dynamometers, and measurements were repeated a week later to check the test-retest reliability of the Biometrics dynamometer. The researchers demonstrated excellent validity, with ICC between 0.983–0.986 and excellent test-retest reliability, with ICC>0.9 [37]. Similar results were obtained in our own research in the field of inter- and intra-rater reliability, with ICC>0.9. We also carried out a three-time assessment for the right and left hand, but in the assessment of intra-rater reliability we used a longer period of time, i.e. two weeks between the measurements, to provide adequate time to prevent recall bias and ensure that the administration of the instruments across time was independent. The test-retest reliability of the Biometrics E-link evaluation system was also evaluated by Kennedy et al.; however, their study was conducted with participants with rheumatoid arthritis [55]. The researchers showed high levels of test-retest reliability, with ICC >0.9 for the evaluation of one test and the mean of three for pain-free grip strength in participants with rheumatoid arthritis. Based on the results obtained, the authors indicate that one test of pain-free grip strength using the Biometrics E-link evaluation system is reliable and can save valuable clinical time, while reducing the burden associated with the evaluation of patients with rheumatoid arthritis [55].

On the other hand, in the area of the Biometrics E-link pinchmeter, test-retest and inter-rater reliability has only been studied in a group of children aged 7–12 years with unilateral spastic cerebral palsy [50], and we cannot compare our results for healthy participants aged 20–24 years to results for children aged 7–12 years with cerebral palsy. We were unable to find published sources that would allow a comparative discussion of the results. Nevertheless, it can be clearly stated that both in our own research and in research by Dekkers et al. [50], excellent intra- and inter-rater reliability was demonstrated using the Biometrics E-link Pinchmeter to assess pinch strength, despite significant differences in the studied populations in terms of both age and functional efficiency and motor control. It is also worth noting that in our own research measurements were taken with the pinchmeter three times for each hand with three different settings, i.e. key, three jaw chuck, tip-to-tip, and the analysis of the consistency of measurements for all three settings, both for the right and left hand, showed high values of Pearson correlation coefficient close to 1, high ICC values >0.9, and taking into account alternative measurements of consistency with the Cronbach alpha coefficient of >0.9 this indicates a high consistency of measurements between both investigators as well as between tests performed by the same investigator at a time interval. In turn, Dekkers et al. carried out evaluations only for key pinch, which is the easiest in children with unilateral spastic cerebral palsy, and only with this one setting for affected and unaffected hands demonstrated excellent intra-and inter-rater reliability, with ICC>0.9 [50]. On the basis of these results, it can be concluded that the Biometrics E-link pinchmeter is a useful tool for assessing pinch strength and grip strength in healthy participants, taking into account all three pinch types and both hands, and in children with spastic cerebral palsy, taking into account only one pinch type and both hands.

Summing up the above considerations, it can be concluded that grip and pinch measurements performed using the Biometrics E-link EP9 evaluation system are characterised by high consistency and repeatability in the Polish population, and the results of this study confirm that these measurements are easy to perform and can be successfully used in clinical practice.

### Limitations

This study has several limitations. First, it involved healthy young adults aged 20–24 years. However, it has been suggested by researchers that assessment of reliability and validity should be conducted in a group of young and healthy individuals. The evaluation of the tool in a healthy population should be followed with a similar study conducted in a group of patients with neurological disorders adversely impacting the strength of one of the extremities. Moreover, the study was limited to inter- and intra-rater reliability assessment in the field of hand grip and pinch strength. It would be worth conducting further analyses focusing on the assessment of concurrent validity by comparing the device with other tools for hand grip and pinch strength assessment, but also assessing the sensitivity to change in this regard, e.g. produced by a rehabilitation process. Another limitation of the study is linked to the fact that the measurements of grip and pinch strength were conducted without assessment of wrist position sense or the range of motion in the hands and forearm. The latter factors should be considered in further research.

## Conclusions

The measurements made with the Biometrics E-link EP9 evaluation system show high internal and external consistency in hand grip and pinch strength assessment. The Biometrics E-link EP9 can be recommended for daily clinical practice. Further studies are needed to assess the concurrent validity and sensitivity in grip and pinch measurements using the Biometrics E-link EP9 evaluation system.

## Supporting information

S1 Fig Bland-Altman plots showing intra-rater and inter-rater agreement in measurements of strength in the right hand. (DOCX)

S2 Fig Bland-Altman plots showing intra-rater and inter-rater agreement in measurements of strength in the left hand. (DOCX)

S3 Fig Bland-Altman plots showing intra-rater and inter-rater agreement in measurements of finger strength in the right hand. (DOCX)

S4 Fig Bland-Altman plots showing intra-rater and inter-rater agreement in measurements of finger strength in the left hand. (DOCX)

S5 Fig Bland-Altman plots showing intra-rater and inter-rater agreement in measurements of finger strength in the right hand (three jaw chuck). (DOCX)

S6 Fig Bland-Altman plots showing intra-rater and inter-rater agreement in measurements of finger strength in the left hand (three jaw chuck). (DOCX)

S7 Fig Bland-Altman plots showing intra-rater and inter-rater agreement in measurements of finger strength in the right hand (tip to tip). (DOCX)

S8 Fig Bland-Altman plots showing intra-rater and inter-rater agreement in measurements of finger strength in the left hand (tip to tip). (DOCX)

## References

[1] Cai A, Pingel I, Lorz D, Beier JP, Horch RE, Arkudas A. Force distribution of a cylindrical grip differs between dominant and nondominant hand in healthy subjects. Arch Orthop Trauma Surg. 2018;138(9):1323–1331. doi: 10.1007/s00402-018-2997-7 29992376

[2] Baek K, Park JT, Hong J, Kwak K. Hand grip strength for the working-age population in South Korea: Development of an estimation and evaluation model. International Journal of Industrial Ergonomics 2023; 93:103398. doi: 10.1016/j.ergon.2022.103398

[3] Hogrel JY. Grip strength measured by high precision dynamometry in healthy subjects from 5 to 80 years. BMC Musculoskelet Disord. 2015; 16:139. doi: 10.1186/s12891-015-0612-4 26055647 PMC4460675

[4] Vaz M, Thangam S, Prabhu A, Shetty PS. Maximal voluntary contraction as a functional indicator of adult chronic undernutrition. Br J Nutr. 1996;76(1):9–15. doi: 10.1079/bjn19960005 8774213

[5] Norman K, Stobäus N, Smoliner C, et al. Determinants of hand grip strength, knee extension strength and functional status in cancer patients. Clin Nutr. 2010;29(5):586–591. doi: 10.1016/j.clnu.2010.02.007 20299136

[6] Bourdel-Marchasson I, Joseph PA, Dehail P, et al. Functional and metabolic early changes in calf muscle occurring during nutritional repletion in malnourished elderly patients. Am J Clin Nutr. 2001;73(4):832–838. doi: 10.1093/ajcn/73.4.832 11273861

[7] Gupta RK, Mittal RD, Agarwal KN, Agarwal DK. Muscular sufficiency, serum protein, enzymes and bioenergetic studies (31-phosphorus magnetic resonance spectroscopy) in chronic malnutrition. Acta Paediatr. 1994;83(3):327–331. doi: 10.1111/j.1651-2227.1994.tb18105.x 8038539

[8] Padmavathi R, Kurpad AV, Vaz M. Skeletal muscle endurance is reduced in chronically energy deficient adults. Indian J Med Res. 2000; 111:28–34. 10793491

[9] Norman K, Stobäus N, Gonzalez MC, Schulzke JD, Pirlich M. Hand grip strength: outcome predictor and marker of nutritional status. Clin Nutr. 2011; 2:135–42. doi: 10.1016/j.clnu.2010.09.010 21035927

[10] Rantanen T, Avlund K, Suominen H, Schroll M, Frändin K, Pertti E. Muscle strength as a predictor of onset of ADL dependence in people aged 75 years. Aging Clin Exp Res. 2002;14(3 Suppl):10–15. 12475129

[11] Gale CR, Martyn CN, Cooper C, Sayer AA. Grip strength, body composition, and mortality. Int J Epidemiol. 2007;36(1):228–235. doi: 10.1093/ije/dyl224 17056604

[12] Rantanen T, Volpato S, Ferrucci L, Heikkinen E, Fried LP, Guralnik JM. Handgrip strength and cause-specific and total mortality in older disabled women: exploring the mechanism. J Am Geriatr Soc. 2003;51(5):636–641. doi: 10.1034/j.1600-0579.2003.00207.x 12752838

[13] Shiratori AP, Iop Rda R, Borges Júnior NG, Domenech SC, Gevaerd Mda S. Evaluation protocols of hand grip strength in individuals with rheumatoid arthritis: a systematic review. Rev Bras Reumatol. 2014;52:140–147 doi: 10.1016/j.rbre.2014.03.009 24878861

[14] Mahmoud AG, Elhadidy EI, Hamza MS, Mohamed NE. Determining correlations between hand grip strength and anthropometric measurements in preschool children. J Taibah Univ Med Sci. 2020; 1:75–81. doi: 10.1016/j.jtumed.2020.01.002 32110186 PMC7033396

[15] Shechtman O, Gestewitz L, Kimble C. Reliability and Validity of the DynEx Dynamometer, Journal of Hand Therapy 2005;3:339–347 doi: 10.1197/j.jht.2005.04.002 16059855

[16] Incel NA, Ceceli E, Durukan PB, Erdem HR, Yorgancioglu ZR. Grip strength: effect of hand dominance. Singapore Med J. 2002; 5:234–237. 12188074

[17] Fess EE. Guidelines for evaluating assessment instruments. J Hand Ther. 1995; 8:144–148. doi: 10.1016/s0894-1130(12)80312-7 7550625

[18] Benaglia PG, Franchignoni F, Ferriero G, Zebellin G, Sartorio F. Affidabilità e validità dell’ analisi della forza nella presa palmare e nelle pinze in prestazioni isometriche e isocinetiche [Reliability and validity of the analysis of hand grip and pinch force in isometric and isokinetic conditions]. G Ital Med Lav Ergon. 1999;21(1):20–4. 10771714

[19] Kaymak B, Inanici F, Ozçakar L, Cetin A, Akinci A, Hasçelik Z. Hand strengths in carpal tunnel syndrome. J Hand Surg Eur. 2008;33(3):327–31. doi: 10.1177/1753193408090105 18562366

[20] Kyberd PJ, Findlayson D, Jayasuriya M, Chibante F. A Strengthened and Sensorised Custom Silicone Glove for use with an Intelligent Prosthetic Hand. Med Eng Phys. 2022 Sep; 107:103845. doi: 10.1016/j.medengphy.2022.103845 36068046

[21] Shiau YY, Wang JS. The effects of dental condition on hand strength and maximum bite force. Cranio. 1993;11(1):48–54, doi: 10.1080/08869634.1993.11677940 8358807

[22] Kim M, Shinkai S. Prevalence of muscle weakness based on different diagnostic criteria in community-dwelling older adults: a comparison of grip strength dynamometers. Geriatr Gerontol Int. 2017;17(11):2089–2095. doi: 10.1111/ggi.13027 28517036

[23] Vermeulen J, Neyens JC, Spreeuwenberg MD, van Rossum E, Hewson DJ, de Witte LP. Measuring grip strength in older adults: comparing the grip-ball with the Jamar dynamometer. J Geriatr Phys Ther. 2015;38(3):148–153. doi: 10.1519/JPT.0000000000000034 25594521

[24] Fess EE. Grip Strength. Clinical assessment recommendations (2nd ed), American Society of Hand Therapists, Chicago 1992:41–45.

[25] Mohammadian M, Choobineh A, Haghdoost A, Hasheminejad N. Normative data of grip and pinch strengths in healthy adults of Iranian population. Iran J Public Health. 2014; 8:1113–1122. 25927041 PMC4411908

[26] Mathiowetz V, Weber K, Volland G, Kashman N. Reliability and validity of grip and pinch strength evaluations. J Hand Surg Am. 1984; 2:222–226. doi: 10.1016/s0363-5023(84)80146-x 6715829

[27] Hamilton A, Balnave R, Adams R. Grip strength testing reliability. J Hand Ther. 1994;3:163–170. doi: 10.1016/s0894-1130(12)80058-5 7951708

[28] Peolsson A, Hedlund R, Oberg B. Intra- and inter-tester reliability and reference values for hand strength. J Rehabil Med. 2001;1:36–41. doi: 10.1080/165019701300006524 11480468

[29] Myers E, Triscari R. Comparison of the strength endurance parameters for the Baltimore Therapeutic Equipment (BTE) Simulator II and the Jamar Handgrip Dynamometer. Work. 2017; 1:95–103. doi: 10.3233/WOR-172542 28506016

[30] Beaton DE, O’Driscoll SW, Richards RR. Grip strength testing using the BTE work simulator and the Jamar dynamometer: a comparative study. Baltimore Therapeutic Equipment. J Hand Surg Am. 1995; 2:293–298. doi: 10.1016/s0363-5023(05)80029-2 7775773

[31] Bellace JV, Healy D, Besser MP, Byron T, Hohman L. Validity of the Dexter Evaluation System’s Jamar dynamometer attachment for assessment of hand grip strength in a normal population. J Hand Ther. 2000; 1:46–51. doi: 10.1016/s0894-1130(00)80052-6 10718222

[32] Hamilton GF, McDonald C, Chenier TC. Measurement of grip strength: validity and reliability of the sphygmomanometer and jamar grip dynamometer. J Orthop Sports Phys Ther. 1992; 5:215–219. doi: 10.2519/jospt.1992.16.5.215 18796752

[33] Mathiowetz V. Comparison of Rolyan and Jamar dynamometers for measuring grip strength. Occup Ther Int. 2002; 3:201–209. doi: 10.1002/oti.165 12374997

[34] Shechtman O, Davenport R, Malcolm M, Nabavi D. Reliability and validity of the BTE-Primus grip tool. J Hand Ther. 2003; 1:36–42. doi: 10.1016/s0894-1130(03)80022-4 12611444

[35] Conforto I, Samir C, Chausse F, Goldstein A, Pereira B, Coudeyre E. Comparison of psychometric properties between the Labin, a new electronic dynamometer, and the Jamar: Preliminary results in healthy subjects. Hand Surg Rehabil. 2019; 5:293–297. doi: 10.1016/j.hansur.2019.07.009 31386926

[36] Cildan Uysal S, Tonak HA, Kitis A. Validity, reliability and test-retest study of Grip strength measurement in two positions with two dynamometers: Jamar® Plus and K-Force® Grip. Hand Surg Rehabil. 2022; 3:305–310. doi: 10.1016/j.hansur.2022.02.007 35283336

[37] Allen D, Barnett F. Reliability and validity of an electronic dynamometer for measuring grip strength. International Journal of Therapy and Rehabilitation 2011; 185:258–264 doi: 10.12968/ijtr.2011.18.5.258

[38] Huang L, Liu Y, Lin T, et al. Reliability and validity of two hand dynamometers when used by community-dwelling adults aged over 50 years. BMC Geriatr. 2022;22(1):580. doi: 10.1186/s12877-022-03270-6 35840905 PMC9284760

[39] Lima FM, Rodrigues LF, Fernandes M, Bertoncello D. Tracking upper limbs fatigue by means of electronic dynamometry. Motriz: Rev. Educ. Fis. 2015; 2:214–221. doi: 10.1590/S1980-65742015000200013

[40] Jarque-Bou NJ, Vergara M, Sancho-Bru JL, Gracia-Ibanez V, Roda-Sales A. Hand Kinematics Characterization While Performing Activities of Daily Living Through Kinematics Reduction. IEEE Trans Neural Syst Rehabil Eng. 2020; 7:1556–1565. doi: 10.1109/TNSRE.2020.2998642 32634094

[41] Casabona A., Valle MS., Dominante C., Laudani L., Onesta MP., Cioni M. Effects of Functional Electrical Stimulation Cycling of Different Duration on Viscoelastic and Electromyographic Properties of the Knee in Patients with Spinal Cord Injury. Brain Sci. 2020; 1:7. doi: 10.3390/brainsci11010007 33374653 PMC7822482

[42] Stock R, Thrane G, Askim T, Anke A, Mork PJ. Development of grip strength during the first year after stroke. J Rehabil Med. 2019; 4:248–256. doi: 10.2340/16501977-2530 30848829

[43] Lameijer CM, Ten Duis HJ, Vroling D, Hartlief MT, El Moumni M, van der Sluis CK. Prevalence of posttraumatic arthritis following distal radius fractures in non-osteoporotic patients and the association with radiological measurements, clinician and patient-reported outcomes. Arch Orthop Trauma Surg. 2018; 12:1699–1712. doi: 10.1007/s00402-018-3046-2 30317380 PMC6224009

[44] Marks M, Audigé L, Herren DB, Schindele S, Nelissen RG, Vliet Vlieland TP. Measurement properties of the German Michigan Hand Outcomes Questionnaire in patients with trapeziometacarpal osteoarthritis. Arthritis Care Res (Hoboken). 2014; 2:245–52. doi: 10.1002/acr.22124 23982906

[45] Severijns D, Lamers I, Kerkhofs L, Feys P. Hand grip fatigability in persons with multiple sclerosis according to hand dominance and disease progression. J Rehabil Med. 2015;47(2):154–60. doi: 10.2340/16501977-1897 25268997

[46] Taechasubamorn P, Nopkesorn T, Pannarunothai S. Comparison of physical fitness between rice farmers with and without chronic low back pain: a cross-sectional study. J Med Assoc Thai. 2010; 12:1415–21. 21344804

[47] Alizadehkhaiyat O, Fisher AC, Kemp GJ, Vishwanathan K, Frostick SP. Upper limb muscle imbalance in tennis elbow: a functional and electromyographic assessment. J Orthop Res. 2007; 12:1651–7. doi: 10.1002/jor.20458 17600835

[48] Bal E, Pişkin A, Ada S, Ademoğlu Y, Toros T, Kayalar M. Comparison between two mini incision techniques utilized in carpal tunnel release. Acta Orthop Traumatol Turc. 2008; 4:234–7. doi: 10.3944/aott.2008.234 19060516

[49] Martínez-Quintana E, Miranda-Calderín G, Ugarte-Lopetegui A, Rodríguez-González F. Rehabilitation program in adult congenital heart disease patients with pulmonary hypertension. Congenit Heart Dis. 2010; 1:44–50. doi: 10.1111/j.1747-0803.2009.00370.x 20136857

[50] Dekkers K, Janssen-Potten Y, Gordon AM, Speth L, Smeets R, Rameckers E. Reliability of maximum isometric arm, grip and pinch strength measurements in children (7–12 years) with unilateral spastic cerebral palsy. Disabil Rehabil. 2020; 10:1448–1453. doi: 10.1080/09638288.2018.1524522 30623690

[51] https://www.biometricsltd.com/medical-grippinch.htm.

[52] Bohannon RW, Schaubert KL. Test-retest reliability of grip-strength measures obtained over a 12-week interval from community-dwelling elders. J Hand Ther. 2005;18(4):426–428. doi: 10.1197/j.jht.2005.07.003 16271690

[53] Królikowska A, Maj A, Dejnek M, Prill R, Skotowska-Machaj A, Kołcz A. Wrist motion assessment using Microsoft Azure Kinect DK: A reliability study in healthy individuals. Adv Clin Exp Med. 2023;32(2):203–209. doi: 10.17219/acem/152884 36135819

[54] Królikowska A, Kusienicka K, Lazarek E, Oleksy Ł, Prill R, Kołcz A, Daszkiewicz M, Janczak D, Reichert P. A Randomized, Double-Blind Placebo Control Study on the Effect of a Blood Flow Restriction by an Inflatable Cuff Worn around the Arm on the Wrist Joint Position Sense in Healthy Recreational Athletes. J Clin Med. 2023;11;12(2):602. doi: 10.3390/jcm12020602 36675531 PMC9867391

[55] Kennedy D, Jerosch-Herold C, Hickson M. The reliability of one vs. three trials of pain-free grip strength in subjects with rheumatoid arthritis. J Hand Ther. 2010;23(4):384–391. doi: 10.1016/j.jht.2010.05.002 20971419

