# Supplementary material for: The reliability of a Biometrics device as a tool for assessing hand grip and pinch strength, in a Polish cohort–A prospective observational study
Source: PLoS One. 2024 May 23;19(5):e0303648. doi: 10.1371/journal.pone.0303648 (PMC11115248; doi:10.1371/journal.pone.0303648)
Supplement: S1 Fig — (DOCX) [file pone.0303648.s001.docx]

**Exam I Researcher 1-2 Exam II Researcher 1-2**


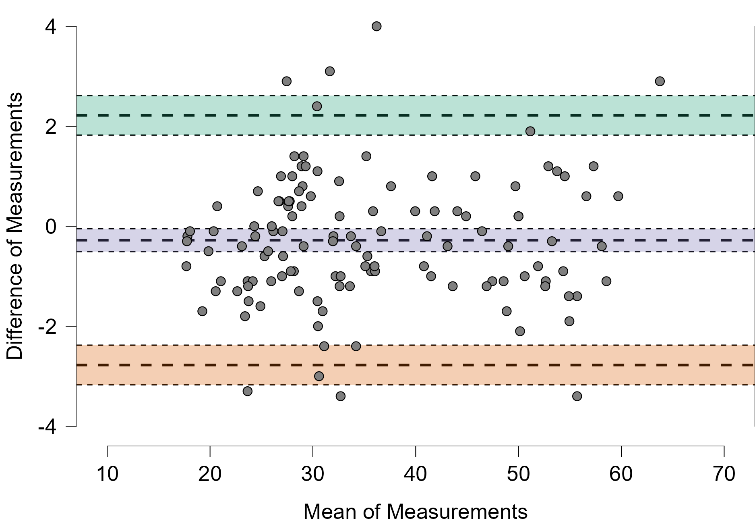

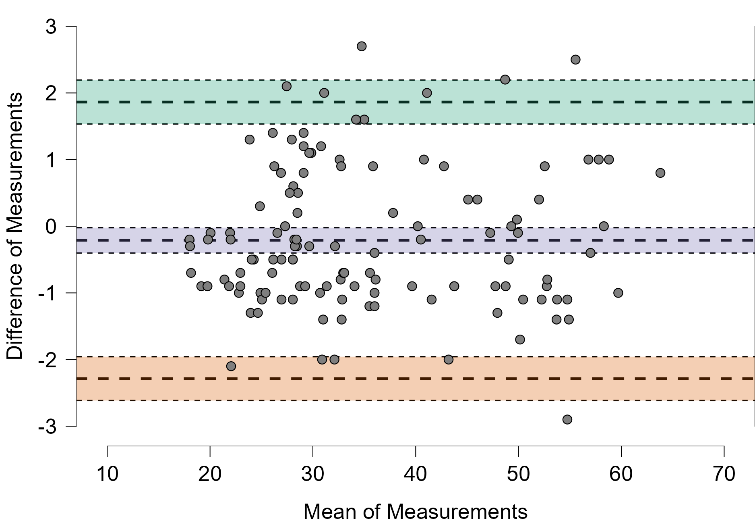


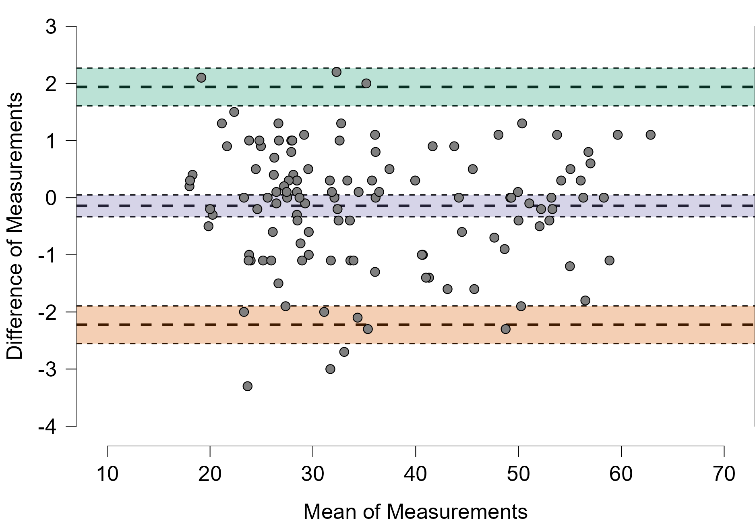

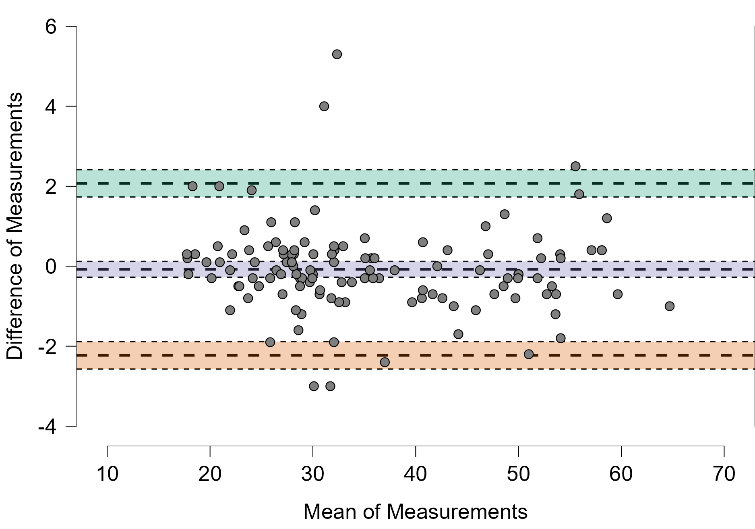
**Exam I-II Researcher 1 Exam I-II Researcher 2**

**S1 Fig. Bland-Altman plots showing intra-rater and inter-rater agreement in measurements of strength in the right hand.**
